# Epigenetic Mechanisms of Escape from BRAF Oncogene Dependency

**DOI:** 10.3390/cancers11101480

**Published:** 2019-10-01

**Authors:** Mehwish Khaliq, Mohammad Fallahi-Sichani

**Affiliations:** 1 Department of Biomedical Engineering, University of Michigan Medical School, Ann Arbor, MI 48109, USA; mehwishk@umich.edu; 2 Program in Cancer Biology, University of Michigan Medical School, Ann Arbor, MI 48109, USA; 3 Department of Dermatology, University of Michigan Medical School, Ann Arbor, MI 48109, USA

**Keywords:** oncogene addiction, BRAF-mutant tumors, BRAF and MEK-targeted therapies, phenotype switching, tumor heterogeneity, epigenetic regulation, adaptive drug resistance, histone-modifying enzymes, chromatin regulation, DNA methylation

## Abstract

About eight percent of all human tumors (including 50% of melanomas) carry gain-of-function mutations in the BRAF oncogene. Mutated BRAF and subsequent hyperactivation of the MAPK signaling pathway has motivated the use of MAPK-targeted therapies for these tumors. Despite great promise, however, MAPK-targeted therapies in BRAF-mutant tumors are limited by the emergence of drug resistance. Mechanisms of resistance include genetic, non-genetic and epigenetic alterations. Epigenetic plasticity, often modulated by histone-modifying enzymes and gene regulation, can influence a tumor cell’s BRAF dependency and therefore, response to therapy. In this review, focusing primarily on class 1 BRAF-mutant cells, we will highlight recent work on the contribution of epigenetic mechanisms to inter- and intratumor cell heterogeneity in MAPK-targeted therapy response.

## 1. Introduction

The discovery of driver oncogenes has revolutionized therapeutic strategies for an increasing number of cancers. These strategies have been primarily focused on either direct inhibition of a mutated oncoprotein, or targeting a component of its effector pathway [1]. The underlying rationale is that tumor cells sustain a state of pathway dependency, or so-called “oncogene addiction”, where cellular growth and survival become highly dependent on the continued activity of the oncogenic pathway [2]. Despite great promise, however, therapeutic inhibition of oncogenic signaling often leads to variable response rates across tumors that carry the same mutated oncogene [3,4,5,6]. A prototypical example of this challenge is observed in cancers that carry alterations in the serine/threonine protein kinase BRAF [7,8]. Oncogenic BRAF alterations lead to hyperactivation of MAP kinase (MAPK) signaling, thereby motivating the development of therapeutic inhibitors of BRAF and downstream MEK kinases [8]. These therapies, however, generate variable cellular and clinical outcomes in BRAF-mutated cancers that originate in distinct tissue types [4,9,10]. For example, they lead to dramatic response rates in melanomas, but their efficacy is limited in thyroid and colorectal carcinomas. Furthermore, despite initially impressive responses [11], BRAF and MEK-targeted therapies ultimately fail to cure most melanomas because of the emergence of therapy resistance [12,13,14,15].

Acquired (late) resistance to BRAF and MEK-targeted therapies involves a diversity of genetic mutations in components of the MAPK pathway [12,16,17,18,19,20,21,22] or parallel signaling networks such as the PI3K/AKT kinase cascade [14,23]. In many cases, however, the emergence of drug-resistant clones cannot be explained by genetic mechanisms. It is thought that drug-resistant clones arise following an initially incomplete and heterogeneous response during the early phase of treatment [24,25,26]. Drug tolerance in this phase could be caused either by enrichment of a subpopulation of undifferentiated or stem-like cells that are intrinsically insensitive to the effect of MAPK inhibition [27,28,29], or by acquisition of adaptive phenotypes with reduced dependency on BRAF and MAPK signaling [30,31,32,33,34,35,36]. Such heterogeneity in the state of BRAF and MAPK dependency appears even among isogenic cells derived from a single clone [35]. Feedback-mediated reactivation of the MAPK pathway or induction of parallel signaling cascades have been recognized as major causes of rapidly induced adaptive resistance to BRAF and MEK-targeted therapies [37,38,39,40,41,42]. Nevertheless, a number of recent studies have identified heterogeneity in lineage program and differentiation state, both among genetically diverse tumors and within genetically homogeneous populations of cells, to be associated with variability in tumor cells’ sensitivity to BRAF and MEK inhibitors [5,35,43,44,45,46].

A major contributor to drug resistance is transcriptome plasticity [47], whereby upon drug treatment, distinct subpopulations of drug-tolerant cells emerge with dynamic and differential gene expression profiles. Epigenetic reprograming following BRAF inhibition can not only induce changes in differentiation state, but also generate new gene expression programs that reduce the cellular requirement for BRAF or MAPK signaling. This phenomenon, referred to as phenotype switching or epigenetic switching, causes fractional response even in genetically homogenous populations of tumor cells. There is still much to learn about how such functionally diverse adaptive phenotypes emerge. However, they appear to be regulated by lineage-dependent, epigenetic mechanisms that continuously reprogram the state of vulnerability in tumor cells [48,49,50,51,52]. These mechanisms complement the genetic code in providing instructions to cells and controlling their responses to internal and external cues in a context-dependent manner. Among these mechanisms are DNA methylation, histone protein modifications, micro(mi)RNAs, and nucleosomal positioning, which together contribute to adopting distinct gene expression states and phenotypes, not only during development and disease but also in response to therapies. The role of only some of these mechanisms has been explored in BRAF-mutant tumors. Nevertheless, a detailed understanding of these mechanisms in BRAF-mutant tumors could be key to increasing the efficacy of MAPK-targeted therapies in these cancers.

In this Review, we discuss the role of epigenetic mechanisms and phenotype plasticity in determining the outcome of MAPK pathway-targeted therapies in BRAF-mutant cancers. We first describe the role of the BRAF kinase, its oncogenic mutations in cancer, and discuss the range of therapies that target BRAF-related malignancies. We then examine the problem of short-lived responses to targeted therapy and the contribution of phenotype plasticity and epigenetic mechanisms to tumor heterogeneity, fractional drug response and drug resistance. We conclude with a discussion of potential therapies with epigenetic inhibitors alone or in combination with MAPK inhibitors to effectively target therapy-resistant BRAF-mutated cancers.

## 2. BRAF Oncogene: Role in MAPK Pathway, Dysregulation in Cancer, and Potential as a Therapeutic Target

The MAPK pathway transduces crucial extracellular signals into intracellular responses, regulating key physiological processes, such as cell proliferation, differentiation and survival. In mammals, the MAPK family consists of four main subgroups, including extracellular-signal-regulated kinase (ERK), MEK4/7-Jun amino-terminal kinase (JNK), stress-activated protein kinase MEK3/6-(p38/SAPK), and MEK5-ERK5 subfamilies [53,54]. The ERK pathway, in particular, is activated by the binding of mitogens to cognate cytokine receptors, G-protein-coupled receptors (GPCRs) or receptor tyrosine kinases (RTKs). Upstream activation of membrane-bound RTKs promotes the recruitment of adaptor proteins, such as the guanine nucleotide exchange factor (GEF), son-of-sevenless (SOS). SOS releases GDP from the RAS proteins (HRAS, KRAS, NRAS), a family of small guanine triphosphatases (GTPases), allowing GTP to bind and activate RAS as a result. Active RAS initiates the three-tier RAF-MEK-ERK cascade. First, active RAS recruits the family of rapidly accelerated fibrosarcoma (RAF) serine/threonine cytosolic kinases (ARAF, BRAF, CRAF) to the plasma membrane, promoting their dimerization [55]. Second, dimerized, catalytically active RAFs directly phosphorylate and activate MEK kinases (MEK1 and MEK2). Third, activated MEK1/2 continue the signaling cascade by phosphorylating and subsequently activating ERK kinases (ERK1 and ERK2) at conserved threonine and tyrosine residues. Activated ERK1/2 kinases phosphorylate several key downstream proteins, including transcription factors and kinases that regulate key cellular processes such as survival, proliferation and differentiation.

Given the importance of the MAPK pathway in maintaining organismal physiology, ensuring a tightly regulated RAS-RAF-MEK-ERK response is vital. In cancer, however, such strictly regulated cellular processes are compromised as a result of MAPK pathway dysregulation. Activating mutations in several components of the MAPK pathway, including RTKs, RAS, RAF, and MEK1/2, have been identified in cancer cells, implicating these modules as oncogenic drivers. In melanomas, for example, malignancy is almost invariably accompanied by mutations that activate this pathway [56]. Among these mutations, BRAF and NRAS are two of the most frequently but mutually exclusively mutated oncogenes with frequencies of >50% and ~30%, respectively. Activating mutations in the MAP2K1 (MEK1) oncogene and loss of the tumor suppressor gene neurofibromin 1 (NF1) are other frequent alterations in melanomas that activate the MAPK pathway, with frequencies of ~6% and ~13%, respectively. Across all human cancers, RAS mutations occur with a frequency of ~15% [57]. MEK and ERK mutations are rare, but almost 8% of all human cancers, including colorectal cancers (10%), papillary thyroid carcinomas (45%), serous ovarian tumors (~30%), non-small cell lung cancers (NSCLCs) (5–10%) and melanomas (>50%) harbor an activating somatic mutation in the BRAF oncogene [57,58,59,60,61,62,63].

BRAF kinase mutations are grouped into three classes. Class 1 BRAF mutations refer to a transversion point mutation in the activation segment of the kinase domain at codon 600, resulting in the substitution of glutamic acid, most commonly with valine (V600E), and sometimes with lysine (V600K), arginine (V600R), or aspartic acid (V600D). The mutated BRAF^V600E^ gene produces a constitutively active oncoprotein, which acts as a monomer independently of upstream signals (i.e., RAS), thereby causing the hyperactivation of the MEK-ERK cascade. Of the BRAF mutations observed in melanomas, the V600E mutation is the most common (~90%) [64]. In addition, BRAF^V600E^ mutations are observed in ~10% of colorectal cancers, 5% of lung adenocarcinomas, 40% of papillary thyroid carcinomas, several glioma subtypes, and a subset of hematological malignancies [58,65,66,67,68]. Class 2 activating BRAF mutations are also RAS-independent, but they function as dimers, resulting in intermediate kinase activity. Non-V600 class 2 mutations have been found in the activation segment (K601E, L597Q), the DFG motif (F595L) as well as the P-loop (G469A) of the BRAF kinase. In contrast, class 3 BRAF mutations (also non-V600) are RAS-dependent and signal as dimers [69,70], with mutations in either the P-loop (G466), catalytic loop (N581) or the DFG motif (D594) [71]. Class 3 BRAF mutants homodimerize with either wild-type BRAF or heterodimerize with CRAF to signal; these dimers have lower kinase activity than their wild-type counterparts [71,72,73]. Mutant forms of BRAF with reduced kinase activity have been observed in lung tumors and melanoma [74,75]. Interestingly, class 3 BRAF mutations often co-occur with upstream mutations in RAS or its negative regulator NF1 [72,76]. Mutations can also occur in ARAF and CRAF genes, albeit at a lower rate [77,78,79]. In addition to point mutations, BRAF gene fusions and in-frame deletions also reveal its characterization as an oncogenic kinase [80,81]. N486-P490-deletion or L485-P490-deletion have been documented in pancreatic, lung, and ovarian cancers [82]. Altogether, these alterations highlight the reliance of multiple cancer subtypes on oncogenic BRAF-MEK-ERK signaling, implicating the BRAF kinase as an exploitable vulnerability and a therapeutic target for such cancers.

## 3. Current Strategies to Target BRAF-Mutated Cancers 

Prior to the identification of BRAF mutations, the first-generation ATP-competitive RAF inhibitor, sorafenib (Nexavar), was developed for targeting RAS-mutant cancers [83]. Sorafenib, however, lacks robustness against cells that harbor BRAF^V600E^ mutations [84]. The discovery of BRAF^V600E^ mutations in multiple cancers spearheaded efforts to target the mutated BRAF. These efforts culminated in the development and FDA approval of second-generation ATP-competitive inhibitors that target class 1 BRAF mutations: vemurafenib (Zelboraf; PLX4032), dabrafenib (Tafinlar; GSK2118436), and encorafenib (Braftovi; LGX818) [7,11,85,86,87]. These inhibitors appear to be selective for BRAF^V600E^ mutated cancers because they efficiently inhibit the monomeric form of RAF (i.e., BRAF^V600E^) that is hyperactivated in these tumors. The stabilization of the structural conformation of αC-helix of the BRAF kinase can determine the effectiveness of RAF inhibitors. For example, binding of an αC-helix-OUT inhibitor molecule, such as vemurafenib, to the first protomer of BRAF stabilizes the αC-helix to an ‘OUT’ conformation; as a result, the second protomer of BRAF is stabilized to an ‘IN’ conformation. This allosteric change in the second protomer prevents binding of a second molecule of BRAF inhibitor [75]. Only at higher concentrations are these αC-helix-OUT BRAF inhibitors able to bind and block both protomers of the BRAF dimer; unfortunately, such extreme concentrations are not feasible for patient treatment. Therefore, αC-helix-OUT inhibitors can inhibit the BRAF^V600E^ kinase, but their impact on cells that lack monomeric BRAF^V600E^ mutations is limited by induction of negative cooperativity and paradoxical activation of ERK signaling [75,88,89,90,91,92,93,94]. Thus, these drugs are not effective against tumors that carry class 2 or 3 BRAF mutations, where BRAF signals as a dimer.

Newer groups of BRAF inhibitors that act as “dimer inhibitors” have also been developed [88,94,95]. LXH254 and LY3009120 are examples of such small molecules that inhibit both mutant BRAF monomers and dimers at similar doses, as they do not induce negative cooperativity at the second site of BRAF dimer. In addition, “paradox breakers” such as PLX8394 are next-generation small molecules that disrupt BRAF-containing dimers, including BRAF homodimers and BRAF-CRAF heterodimers, but not CRAF homodimers or ARAF-containing dimers [96,97]. Since PLX8394 does not inhibit wild-type CRAF dimers and has a wide therapeutic window, it is an attractive candidate for the treatment of tumors driven by class 1 and 2 BRAF mutants and BRAF fusions.

To improve patient response, BRAF inhibitors vemurafenib, dabrafenib and encorafenib have been used in combination with small molecule inhibitors of the MEK kinases, such as cobimetinib (Cotellic), trametinib (Mekinist), and binimetinib (Mektovi). All three compounds are allosteric inhibitors that bind to the MEK kinase and induce conformational changes that subsequently block its kinase activity [98,99]. MEK inhibitors inhibit ERK signaling in both BRAF-mutant and BRAF-wildtype cells. The combination of BRAF and MEK inhibitors overcomes the paradoxical activation of ERK signaling with BRAF inhibitors, while also lowering the toxicities associated with the effect of MEK inhibitors in BRAF-wildtype cells [90,100]. As a result, combination therapy with MEK inhibitors (such as trametinib) and class 1 BRAF inhibitors (such as dabrafenib) have become FDA-approved standard of care for patients with metastatic BRAF-mutated melanomas. BRAF^V600E^ melanoma patients have demonstrated improved response rates with combination MAPK therapy [101]. Unfortunately, however, the clinical benefit of such therapies is still short-lived in the great majority of patients due to the emergence of resistance [19,21].

## 4. Limitations of Current Therapies Targeting BRAF-Mutated Cancers

**Figure 1:**
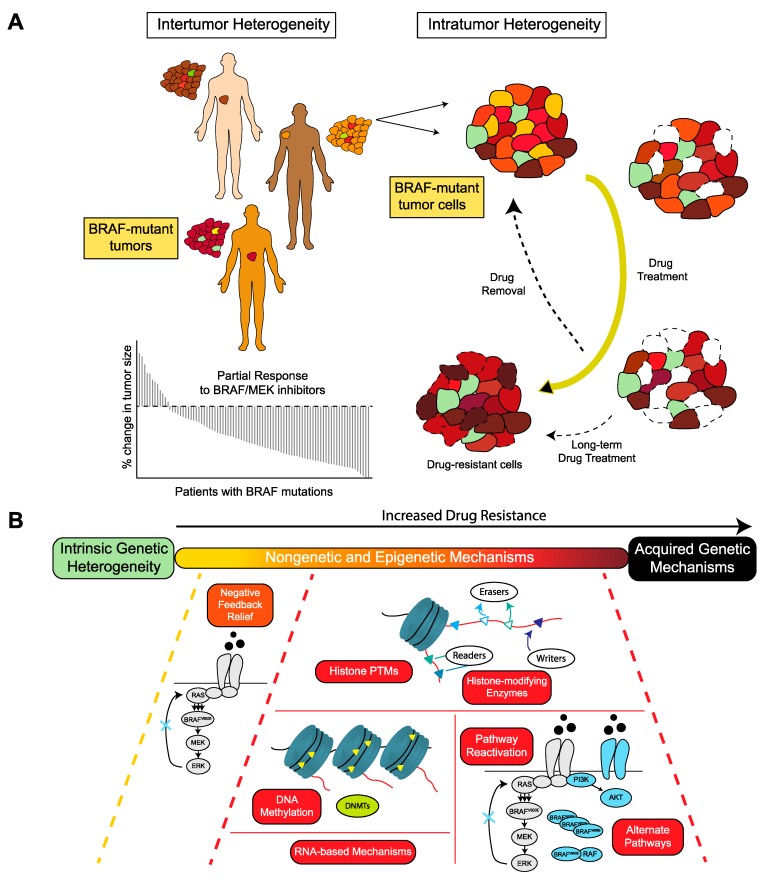
Dynamic mechanisms of inter- and intratumor heterogeneity. (**A**) Intertumor heterogeneity in patients with BRAF-mutant tumors results in a partial response to BRAF and MEK inhibitor therapy. Intratumor heterogeneity in BRAF-mutant tumors comprises of cells at distinct transcriptional cell states due to genetic (green), non-genetic (e.g. pathway rewiring) and epigenetic mechanisms (gradient from yellow to brown). A cell’s BRAF or MAPK dependency can determine its ability to tolerate or adapt to BRAF or MAPK inhibitor therapies. A cell with higher BRAF dependency is more sensitive to short-term BRAF inhibitor therapy (dashed outline), whereas a cell with lower BRAF dependency can better tolerate the drug over time by relying on non-genetic and epigenetic mechanisms. Long-term drug treatment can result in a stable, drug-resistant phenotype, whereas adaptive drug resistance is reversible. (**B**) Upon drug treatment, drug-tolerant cells can employ non-genetic (relief of MAPK negative feedback, MAPK pathway reactivation, and alternate pathways, such as the PI3K pathway) and epigenetic mechanisms (DNA methylation, histone modifications, histone-modifying enzymes, and RNA-based mechanisms) to escape drug effect. Over time, these drug-tolerant, drug-adaptive mechanisms can ensure cells survive long enough to acquire advantageous mutations that lower the cell’s BRAF dependency and lead to long-term resistance.

Despite substantial advances in the development of therapeutics focused on MAPK pathway inhibition in BRAF-mutated cancers, resistance and subsequent tumor relapse remain a major challenge to the durable success of these therapies [16]. For example, despite the initial rapid response rates with BRAF and MEK inhibitor combinations in melanoma patients, resistance typically emerges within the first two years of therapy [3,102]. Intrinsic and acquired resistance to MAPK inhibitors involves a diversity of genetic, non-genetic and epigenetic factors (Figure 1).

Genetic mechanisms of resistance comprise of mutations in the MAPK pathway, including NRAS, KRAS and MEK1 mutations, NF1 loss of function, as well as mutations in the AKT/PI3K pathway, such as AKT mutations and loss of PTEN [16,103,104,105,106]. Additional mechanisms of resistance include those that result from overexpression of RTKs (PDGFRB, IGF-1R, EGFR), COT (MAP3K8) kinase, amplification of BRAF or dimerization of alternatively-spliced BRAF isoforms [16,17,107,108,109,110]. In addition, treatment with BRAF and MEK inhibitors leads to relief of feedback inhibition of MAPK signaling. In BRAF^V600E^ cancer cells, hyperactivated ERK signaling results in feedback suppression of upstream factors, such as SOS and RTKs, thereby inhibiting RAS activity. When BRAF signaling is pharmacologically inhibited, ERK-mediated feedback is relieved, RAS-GTP levels increase, and subsequently a variety of RAS-mediated signaling pathways get activated [37,111]. By relieving the negative feedback regulation of the MAPK pathway, BRAF and MEK inhibitors attenuate their maximal efficacy through the induction of adaptive resistance mechanisms. Such resistance is often explained by activation of pro-growth pathways, including both cell-autonomous mechanisms, such as up-regulation or rewiring of mitogenic signaling cascades, and non-cell-autonomous changes in the microenvironment. Studies of adaptive responses in BRAF-mutated cancers have shown that these responses are remarkably diverse [38]. They involve different combinations of ERK pathway reactivation and induction of parallel signaling cascades such as the PI3K/AKT, JNK/c-Jun and NF-κB pathways, with the net effect of reducing drug efficacy. The breadth and complexity of these responses, however, have challenged traditional approaches of developing unified schemes to prevent BRAF and MEK inhibitor therapy resistance in BRAF-mutant tumors.

## 5. Epigenetic Mechanisms of MAPK Dependency in BRAF-Mutated Tumors

The term “epigenetic” generally refers to non-genetic, heritable mechanisms of gene regulation that contribute to cell fate and phenotype. In such cases, cellular phenotypes are governed by mechanisms that control differential gene expression profiles. Mechanisms of epigenetic regulation include DNA methylation, mRNA processing, micro(mi)RNAs, RNA stability, nucleosomal positioning and occupancy, and chromatin remodeling [112]. Key components involved in shifting chromatin architecture and providing transcription factors access to relevant genes involve DNA methylation and post-translational modifications (PTMs) on core histone proteins. These modifications allow chromatin to exist in an active (i.e., open) or inactive (i.e., closed) state, corresponding with either promoting or inhibiting transcription of relevant genes. In cancer, epigenetic remodeling can promote a dedifferentiated or progenitor-like state, aiding cells with oncogenic mutations and helping to promote tumorigenesis.

Epigenetic mechanisms (together with genetic factors) contribute to both inter- and intratumor heterogeneity. Intertumor heterogeneity occurs between patients diagnosed with tumors sharing the same histology. Intratumor heterogeneity, on the other hand, occurs in tumor cells within the same patient. As a result of such heterogeneity, different subpopulations of tumor cells with a range of epigenetic states may dynamically co-exist within the same tumor. The baseline epigenetic state of a tumor cell is established by its developmental lineage and differentiation state [113,114]. However, microenvironmental perturbations, such as drug treatment or unfavorable metabolic conditions, compel a cell to shift its epigenetic state by inducing modifications in the chromatin structure, modulating gene expression profiles and adjusting its proteomic state. Changes in the proteomic state, as influenced by epigenetic modifications, determine phenotypic consequences by regulating the adaptive or compensatory signals initiated by cells to ensure cell survival. Overall, these mechanisms, present intrinsically or employed by the cancer cells following drug treatment, can lead to their fractional responses to therapies [32,115,116,117,118]. They nonetheless provide new therapeutic vulnerabilities that could be exploited to combat drug resistance [119].

In the case of BRAF-mutant melanomas, tolerance to BRAF and MEK kinase inhibitors is associated with distinct phenotype plasticity of melanoma differentiation state and global alterations in gene expression programs [5,26,120]. These programs include: (i) a pigmented melanocytic state associated with transcription factor MITF, a key regulator of melanocyte lineage [121,122,123], (ii) a neural crest-like state characterized by NGFR and markers of the neural crest, a melanocyte precursor [35,124,125], and (iii) an undifferentiated state characterized by low levels of the transcription factor SOX10 and high expression of receptor tyrosine kinases such as AXL and EGFR [44,45]. Despite the recurrent composition of differentiation states among melanomas, the frequency of individual states appears to vary from one tumor to the next [46]. Such heterogeneity could influence the nature and relative strength of the pathways through which each tumor responds or adapts to BRAF and MEK inhibition [126]. At the single-cell level, transcriptional heterogeneity and epigenetic reprogramming enable drug resistance in BRAF^V600E^ melanoma cells. Prior to MAPK inhibitor treatment, cells display transcriptional variability with transient fluctuations in resistance genes. This phenomenon allows cells to transition between a non-resistant and pre-resistant phenotype. Upon drug addition, epigenetic reprogramming causes the generation of transiently heritable resistance phenotypes [47]. Long-term drug treatment can ultimately enrich drug-adapted cells with additional mutations that permit cell survival and lead to stable drug resistance [51].

Although both genetic and epigenetic factors contribute to drug resistance, the intricacies of the interplay between the two groups of mechanisms and their control of plasticity and tumor heterogeneity in BRAF-mutant cancers remains to be fully elucidated. What is clear is that they need not be mutually exclusive. Through the emergence of a drug-tolerant, dedifferentiated, slow-cycling cell subpopulation, BRAF-mutant melanomas treated with MAPK inhibitors adapt to drug over time. During the adaptation process, alterations in the transcriptome or epigenome may be transient and reversible upon drug removal [35], but continuous drug treatment can stabilize these transcriptionally and epigenetically-altered phenotypic states [127]. Epigenetic mechanisms that regulate gene expression and phenotypic states may include DNA methylation, histone-modifying enzymes and histone modifications.

## 6. DNA Methylation in BRAF-Mutated Cancers

In eukaryotes, DNA methylation is a process by which methyl groups (-CH3) are covalently transferred to the cytosine bases of DNA. The transfer process is catalyzed by three specific enzymes called DNA methyltransferases (DNMTs). DNMTs use the methyl donor, S-adenosyl methionine (SAM) to transfer a methyl group to the 5-carbon of cytosine, producing 5-methylcytosine (5-mC). Additional modifications of cytosines include 5-formylcytosine (5-fC), 5-hydroxymethylcytosine (5-hmC), and 5-carboxylcytosine (5-caC) at CpG sites [128,129,130,131]. CpG sites or dinucleotide regions refer to a portion of the genome with a cytosine base preceding a guanine base with the ‘p’ denoting a phosphodiester bond that is shared between the dinucleotides. CpG sites can be found in coding regions of genes and are usually methylated. CpG islands refer to short DNA sequences that are mainly CG-rich (60%) and remain unmethylated. CpG islands are usually found in gene promoters or transcription start sites of either genes poised for transcription or genes that are highly transcribed [132]. On the other hand, CpG shores, which refer to regions of the DNA that surround CpG islands, contain fewer CG dinucleotides and correlate with gene expression [133,134].

Hypomethylation, defined as the absence or loss of the methyl group from cytosines, can open up the tightly-packed chromatin, leading to genomic instability and chromosomal abnormalities, such as translocations and deletions [135]. Hypermethylation, in contrast, can lead to transcriptional repression. Approximately 80% of the CpG sites in the human genome are methylated [136]. Cancer cells display decreased levels of 5-methylcytosine [137] as well as global DNA hypo- or demethylation and de novo site-specific hypermethylation. About 5–10% of CpG islands at promoters, which are normally unmethylated, become hypermethylated in many cancers [138]. These genes code for tumor suppressors and DNA repair proteins [139,140]. Of the 8–15% of colorectal cancers with an activating BRAF^V600E^ mutation, most have been found to contain genome-wide hypermethylated CpG islands, termed CIMP for CpG Island Methylator Phenotype [141,142,143]. In a study of serrated polyps and colorectal cancers, the CIMP-high cancers were more frequently identified with the BRAF^V600E^ mutation, implying a significant association between DNA methylation and BRAF mutational status [144]. Although there is a strong association between CIMP and colorectal cancers harboring BRAF^V600E^ mutations, the relationship between CIMP and BRAF^V600E^ melanomas is less clear. In cutaneous melanoma, TCGA analysis revealed a lower prevalence of BRAF hot-spot mutations in CIMP clusters [56]. Moreover, comparison of BRAF^V600E^ and BRAF^WT^ melanomas revealed no significant difference in the number of hypermethylated peaks [145]. These differences hint at the differential contribution of tissue-specific factors and alternative gene dependencies and programs within each tumor type. Interestingly, the pathway regulating hypermethylation of CIMP genes in BRAF^V600E^ colorectal cancers was also found to be important in epigenetic silencing in BRAF^V600E^ melanomas. This hypermethylation process was mediated by the transcriptional repressor, v-maf avian musculoaponeurotic fibrosarcoma oncogene homolog G (MAFG). BRAF^V600E^-mediated ERK activation resulted in phosphorylation and subsequent preservation of MAFG levels, leading to an increase in MAFG-bound DNA regions. Following DNA binding, MAFG recruited co-repressor molecules, including DNMT3B, BACH1 and CHD8; this co-repressor complex was involved in hypermethylating and silencing the promoter regions of CIMP genes [146].

Besides aberrant DNA methylation marks, mutations in the DNMT enzymes have also been identified in cancer. DNMT3B, considered a de novo DNA methyltransferase for depositing and maintaining methyl marks, has been implicated in tumor progression [147,148]. DNMT3B expression is upregulated in melanoma and is associated with reduced patient survival. In a genetically engineered BRAF^V600E^, PTEN knockout melanoma mouse model, DNTM3B loss delayed melanoma formation and increased animal survival [149]. In drug-tolerant BRAF^V600E^ melanoma cells that emerged following MAPK inhibitor treatment, DNMT3A, DNMT3B, and DNMT1 were differentially expressed, resulting in low global DNA methylation levels [150]. An integrated epigenetic analysis of human tumors treated with MAPK inhibitors identified drug resistance programs corresponding with transcriptomic and methylomic alterations [15]. Transcriptomic analysis revealed differential mRNA expression in genes such as c-MET, LEF1 and DUSP4 in MAPK inhibitor-resistant tumors. Such gene expression patterns correlated with differential methylation at CpG clusters, suggesting key connections between epigenetic regulation of DNA methylation patterns, gene expression profiles and MAPK inhibitor resistance.

## 7. Histone-Modifying Enzymes and Post-Translational Modifications in BRAF-Mutated Cancers 

In eukaryotic cells, the basic structural unit of chromatin, termed nucleosome, consists of DNA wrapped around an octamer of four core histone proteins. A histone octamer is comprised of two copies each of four histone proteins, including H2A, H2B, H3, and H4. A linker histone, H1, functions to stabilize the nucleosome structure. The N-terminal tails of these histone proteins can be covalently and reversibly altered with a diverse array of post-translational modifications (PTMs), including methylation, acetylation, glycosylation, phosphorylation, ADP-ribosylation, crotonylation, citrullination, sumoylation, and ubiquitylation. These dynamic histone PTMs and the specific histone protein that is modified can promote either transcriptional activation or repression of applicable genes by remodeling the chromatin structure. Chromatin remodelers include histone-modifying enzymes (HMEs), which are categorized into three main groups: (a) readers, enzymes that recognize histone PTMs and facilitate recruitment of protein complexes; (b) erasers, enzymes that remove a histone PTM; and (c) writers, enzymes that deposit the histone PTMs. HMEs that establish histone PTMs include, but are not limited to, histone methyltransferases and histone acetyltransferases (HATs), whereas HMEs that erase PTMs include histone demethylases and histone deacetylases (HDACs). HMEs function in complexes (i.e., Polycomb and Trithorax) and regulate gene expression by facilitating either the opening of condensed chromatin to allow transcriptional machinery access to specific promoters, or the compacting of open chromatin to prevent relevant genes from being transcribed. Besides modifying residues on the histone tails, HMEs can also target non-histone proteins for regulation, such as p53, Rb and Myc. Together, the combination of histone PTMs, constituting the “histone code”, as well as the presence of bivalent histone PTMs, referring to promoters possessing both activating and repressing marks, reveal the complexity of chromatin regulation and remodeling [151,152].

The global pattern of histone PTMs have been used to predict clinical outcome in prostate cancer recurrence, which is indicative of the close relationship between histone PTMs and disease [153]. Changes in the global levels of histone PTMs and mutations in HMEs have also been linked to additional cancers [154,155,156]. In a zebrafish model of BRAF^V600E^ and p53-mutated melanoma, several genes were found to cooperate with BRAF^V600E^ to accelerate melanomagenesis. SETDB1, a lysine methyltransferase that functions by depositing a methyl mark on histone H3 lysine 9 (H3K9), was found to increase melanoma formation [157]. In addition to lysine methyltransferases, two structurally unrelated H3K9 lysine demethylases, LSD1 and JMJD2C cooperate with BRAF to overcome oncogene-induced senescence (OIS) and contribute to the development of malignant melanoma [48,158]. Whereas BRAF (or RAS) expression in premalignant melanocytic nevi leads to OIS–which is characterized by cell-cycle arrest and an impediment of tumor formation–the overexpression of JMJD2C or LSD1 in BRAF-mutated cells promotes melanomagenesis by overcoming the dynamic phenotypic state enabled by BRAF-induced senescence. BRAF-mutated cells overcome OIS by utilizing epigenetic reprogramming to their advantage in promoting tumor formation. Such opportunistic methods employed by BRAF-mutant cells also expose their therapeutic vulnerabilities, as evidenced by the sensitivity of BRAF-mutated melanoma cells to LSD1 and JMJD2 inhibitors. These findings are especially powerful since epigenetic dependencies (LSD1 or JMJD2C) in melanoma are not associated with sensitivity to BRAF inhibitors, implicating a potential therapeutic approach for patients relapsing on BRAF-targeted therapies.

Changes in expression levels or activities of key histone-modifying enzymes (HMEs) have been associated with phenotype plasticity and drug tolerance in a variety of cancers. In EGFR-mutant non-small cell lung cancer (NSCLC) cells treated with EGFR inhibitor gefitinib, a reversibly drug-tolerant persister phenotype was reported to be dependent on JARID1A (KDM5A), a Jumonji histone demethylase that removes methyl groups from histone H3 lysine 4 (H3K4) [32]. Similarly, exposure of BRAF^V600E^ melanoma cells to sublethal levels of stress-inducing conditions, such as BRAF and MEK inhibitors, induced the surviving cells to adopt a less differentiated, drug-tolerant state, with elevated expression of histone demethylases such as KDM6A, KDM6B, KDM1B, JARID1A, and JARID1B [28,29,31]. These modifications were concomitant with increased levels of H3K9me3 and lower levels of H3K4me3 and H3K27me3, indicating epigenetic activation and silencing of select genes [31]. Several histone methyltransferases, such as SETDB1 and SETDB2, have also been shown to be upregulated in response to BRAF and MEK inhibition while knockdown of these HMEs restored drug sensitivity [150]. In additional studies of BRAF^V600E^ melanoma cells that were resistant to MAPK inhibitors, expression of the histone deacetylase SIRT6 was shown to be downregulated [116]. Interestingly, only partial, but not complete loss of SIRT6 (i.e., haploinsufficiency) conferred resistance to BRAF and MEK inhibitors. In this case, SIRT6 haploinsufficiency led to upregulation of the IGF-1 receptor (IGF-1R) and subsequent AKT pathway activation.

## 8. Alternative Combinatorial Treatments to Overcome Therapy-Resistant BRAF-Mutated Cancers

Acquired resistance, due to intratumor heterogeneity, remains an obstacle for the complete eradication of tumor cells. The emergence of reversible drug-tolerant persister (DTP) cells may lead to fully resistant clones. However, these DTP cells also possess novel and exploitable vulnerabilities. For example, cancer cells that lose epithelial markers and obtain a high-mesenchymal cell state not only correlate with the ability to become therapy-resistant [159], but also have increased vulnerability to compounds inducing ferroptosis and statins [115,118]. Specifically, these compounds are more effective in cancer cells with a high-mesenchymal state due to dependency on the lipid glutathione hydroperoxidase, GPX4. If GPX4 is inhibited, the DTP cells, which rely on GPX4, undergo ferroptotic cell death [115,118].

**Figure 2:**
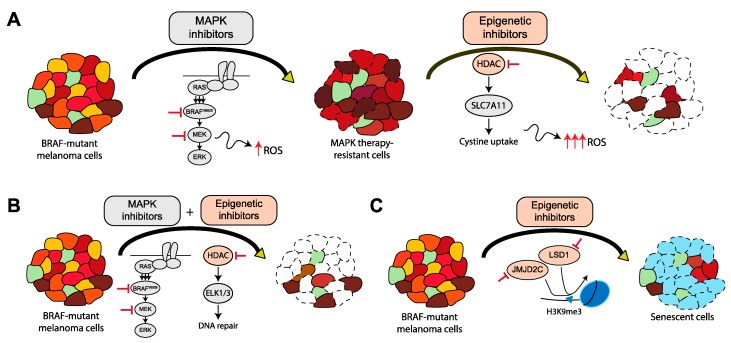
Strategies to target epigenetic mechanisms of drug resistance in BRAF-mutant melanoma cells. (**A**) BRAF-mutant melanoma cells with acquired resistance to MAPK inhibitor therapy are vulnerable to HDAC inhibitors, which suppress the transporter, SLC7A11, and increase reactive oxygen species (ROS) to lethal levels, causing apoptosis. (**B**) Treatment with a combination of MAPK and HDAC inhibitors is effective in inducing apoptosis in BRAF-mutant melanoma cells. The combination treatment reduces ELK1 and ELK3 levels, resulting in aberrant activity of DNA repair mechanisms and subsequent melanoma cell death. (**C**) Treatment of BRAF-mutant melanoma cells with LSD1 or JMJD2C inhibitors reduces melanoma cell growth and restores oncogene-induced senescence.

In a similar vein, other vulnerabilities of therapy-resistant cancer cells need to be examined and exploited to harness a more complete and stable response to therapy. In BRAF-mutant melanoma and NSCLC cells, the YAP protein, a component of the Hippo tumor-suppressor pathway, is an important mediator of resistance to MAPK-inhibitor therapy. Sensitivity of BRAF-mutant cells to MAPK inhibitors increases upon YAP inhibition [160,161]. Drug resistance in YAP-overexpressing BRAF-mutant melanoma cells has been mediated by BET bromodomain proteins. Inhibition of BET proteins in drug-resistant cells reverts these resistant cells to a sensitive state and impairs tumor cell viability [162]. In BRAF^V600E^ melanomas, combination treatment inhibiting BET bromodomains and either BRAF^V600E^ or MEK kinases synergistically promote melanoma cell death and block cell proliferation [35,163,164]. BRAF^V600E^ melanoma cells with acquired resistance to BRAF/MEK inhibitors have been shown to exhibit increased production of ROS [127,165]. As a result of high ROS production, drug-resistant cells acquire susceptibility to HDAC inhibitors, which inhibit the cystine/glutamine transporter, SLC7A11, further increasing the ROS levels to lethal quantities (Figure 2A) [127]. While this study highlights the importance of sequential treatment of MAPK and HDAC inhibitors in combating MAPK inhibitor-resistant cells, another study found the simultaneous combination of MAPK inhibitors and Class 1 HDAC3 inhibitor, entinostat, was able to suppress melanoma tumor growth (Figure 2B) [166]. Besides HDACs, pharmacological inhibition of H3K9 demethylases alone or in combination with MAPK inhibitor therapy may also provide benefit for the treatment of BRAF^V600E^ melanomas (Figure 2C) [48].

In addition to histone erasers and readers, histone writers are also dysregulated in cancer. A component of the Polycomb Repressive Complex 2 Subunit (PRC2), EZH2 is a histone lysine methyltransferase, which catalyzes trimethylation of H3K27. In melanoma, prostate and breast cancer, EZH2 is overexpressed [167]. When EZH2 is inhibited in melanoma, both cell proliferation and the invasive potential of melanoma cells is reduced [168]. In addition, the transcription factor, NFATc2, is upregulated in melanoma and its inhibition suppresses EZH2 levels. BRAF^V600E^ cell lines, which have intrinsic resistance to vemurafenib, exhibited a marked increase in cell death upon the combination treatment of both NFATc2 and EZH2 inhibitors [169].

The task of identifying novel therapies is multi-faceted and future work requires an in-depth understanding of epigenetic mechanisms, tumor heterogeneity, and drug resistance. This involves elucidating how each component influences or interacts with each other to mediate cellular response and how these data can be applied to unveil novel therapeutic strategies for improving clinical outcomes.

## 9. Conclusions

Therapy-induced acquired resistance to BRAF and MAPK inhibitors relies on the cooperative communication between genetic, non-genetic and epigenetic mechanisms. Following drug treatment, biochemical and epigenetic reprogramming can dynamically regulate gene expression profiles, leading to complex and fluctuating phenotypic states. These phenotypically plastic cells are selected upon MAPK inhibitor treatment, demonstrating a loss of BRAF dependency in drug-tolerant cells. 

Research into the epigenetic mechanisms of BRAF dependency or escape from MAPK pathway dependency in BRAF-mutated cancers is still in its infancy. Progress has been made in understanding transcriptional programs associated with the differentiation states of BRAF-mutated cancers and MAPK inhibitor resistance, but we are only beginning to uncover how differentiation states are regulated and how their dynamic fluctuations can lead to drug tolerance. Systems biology approaches, combining single-cell and molecular analyses, can help to elucidate the contribution of epigenetic states, reprogramming and cellular response in determining BRAF dependency. More importantly, in MAPK inhibitor-resistant cells, the discovery of acquired epigenetic dependencies holds great promise for the development of new, effective therapies.
